# Clinical Utility of Serum Cystatin C in Predicting Diabetic Distal Sensorimotor Polyneuropathy

**DOI:** 10.3390/biomedicines14030544

**Published:** 2026-02-27

**Authors:** Reem M. Alhammad, Abdulaziz Alshoumar, Jehad Alorainy, Hana Albulaihe, Mohammed Mujammami, Marwah Alrehaili, Mohammad I. Awan

**Affiliations:** 1 Department of Internal Medicine, College of Medicine, King Saud University, Riyadh 14611, Saudi Arabia; aalshoumar@ksu.edu.sa (A.A.); jehadorainy@gmail.com (J.A.); halbulaihi@ksu.edu.sa (H.A.); mhmujammami@ksu.edu.sa (M.M.); malrahili@moh.gov.sa (M.A.); miawan@ksu.edu.sa (M.I.A.); 2 Department of Internal Medicine, Ohud Hospital, Ministry of Health, Madina 42354, Saudi Arabia

**Keywords:** cystatin C, diabetic distal symmetric sensorimotor polyneuropathy, distal symmetric polyneuropathy, diabetic neuropathy, type 2 diabetes mellitus

## Abstract

**Background**: Approximately half of patients with diabetes mellitus (DM) develop diabetic distal symmetric sensorimotor polyneuropathy (DM-DSPN), yet no reliable biomarkers for its early detection exist. This study assesses cystatin C (CysC), a naturally occurring protein, in diabetic persons with and without large-fiber DM-DSPN. **Methods**: This study involved persons with diabetes (HbA1c > 6.5%) visiting specialized diabetic clinics at King Saud University Medical City (KSUMC) in Riyadh, Saudi Arabia. Clinical features, laboratory data, nerve conduction findings, and serum CysC levels were assessed. DM-DSPN was diagnosed if signs of large nerve fiber impairment were present in the lower extremity in a symmetric and length-dependent pattern. Participants were designated as diabetic with or without large-fiber DSPN (+DM/+DSPN and +DM/−DSPN, respectively) based on validated composite scores of nerve conduction attributes. **Results**: A total of 52 persons with diabetes were included for analysis (24 with +DM/+DSPN and 28 with +DM/−DSPN). One participant had type 1 DM; all remaining participants had type 2 DM. In multivariate regression, serum CysC ≥ 0.88 mg/L was significantly associated with DM-DSPN. Serum CysC was significantly associated with peroneal and ulnar compound muscle action potential amplitudes (*p*-value = 0.003 and *p*-value = 0.03, respectively) and peroneal and tibial motor nerve conduction velocities (*p*-value = 0.009 and *p*-value = 0.0003, respectively). **Conclusions**: Serum CysC levels > 0.9 mg/L are associated with DM-DSPN (86% sensitivity and 81% specificity), independently of HbA1c or GFR. Serum CysC is also associated with peroneal and ulnar compound muscle action potential amplitudes and peroneal and tibial motor nerve conduction velocities. Larger studies are needed to determine the role of CysC as a potential biomarker of DM-DSPN.

## 1. Introduction

Diabetic neuropathy is the most prevalent complication of diabetes, affecting approximately 40% of patients with diabetes during the course of their disease [1,2]. Diabetic distal symmetric sensorimotor polyneuropathy (DM-DSPN) is the most common form of diabetic neuropathy [3,4]. Without large-scale intervention, an estimated 783 million individuals globally will develop diabetes by 2045, half of whom are expected to develop DSPN [5].

DM-DSPN is an irreversible condition that predisposes patients to a myriad of complications, such as foot ulcers, gangrene, and non-traumatic amputations [6]. The annual cost of diabetic neuropathy and its associated complications is more than USD 10 billion in the United States alone [7].

The physical and mental impacts experienced by patients with DM-DSPN, as well as the significant social and economic burden of this condition, highlight the urgent need to identify early biomarkers of DM-DSPN. Moreover, no specific therapies exist, and glycemic control remains the sole intervention for DM-DSPN prevention. Thus, the identification of DM-DSPN biomarkers could strengthen our knowledge of the pathological mechanisms underlying this condition, potentially guiding the development of more effective therapies.

Cystatin C (CysC) is a low-molecular-weight protein that belongs to the cystatin superfamily and functions as a potent cysteine protease inhibitor [8]. It regulates enzymatic activity by blocking cathepsins, a group of proteases involved in protein degradation and cellular homeostasis. CysC is ubiquitously expressed in bodily fluids, including cerebrospinal fluid (CSF), blood, and saliva, and plays roles in both physiological and pathological conditions such as neurodegeneration and neuroinflammation [9].

Moreover, a previous study of approximately 900 persons with diabetes revealed that serum CysC was associated with a high risk of developing DM-DSPN after controlling for age, diabetes duration, hemoglobin A1c (HbA1c), creatinine (Cr), urinary albumin, and the glomerular filtration rate (GFR) [10]. However, to our knowledge, no studies have evaluated the association between serum CysC and DM-DSPN using objective, validated, and referenced criteria for DM-DSPN definition and severity staging. Furthermore, the association of CysC with various nerve conduction (NC) attributes has not been reported in the literature.

Therefore, in this study, we evaluate the clinical utility of serum CysC in DM-DSPN as a potential biomarker using validated criteria for DM-DSPN severity staging. We also assess the association between CysC and NC attributes. Our findings are exploratory but can guide larger studies assessing the role of serum CysC in DM-DSPN development and progression.

## 2. Methods

### 2.1. Setting and Clinical and Laboratory Features

This cross-sectional study was conducted at King Saud University Medical City (KSUMC) in Riyadh, Saudi Arabia, between January 2021 and January 2024. Adult persons with type 1 or type 2 diabetes visiting the diabetic specialty clinics at KSUMC were invited to participate in the study. All research participants signed informed consent forms approved by our institutional review board.

Participants were included if they had documented HbA1c higher than 6.5% at any time prior to enrollment in the study. Participants with documented causes of peripheral neuropathy other than diabetes, such as vasculitides, nutritional deficiencies, infiltrative tumors, inherited neuropathies, neoplastic or inflammatory dermatologic conditions, or exposure to neurotoxic medications (e.g., chemotherapy, isoniazid), or a prior history of bariatric surgery, were excluded.

Hypertension was defined as systolic blood pressure (SBP) > 140 mmHg, diastolic blood pressure (DBP) > 90 mmHg, documented hypertension, or the use of antihypertensive medications. Anthropometric variables such as height, weight, and waist circumference were measured for all research participants. Waist circumference—iliac crest (WC-IC) was measured in the horizontal plane at the superior border of the right iliac crest. Waist circumference—mid-abdomen (WC-mid) was measured in the horizontal plane midway between the lowest rib and the iliac crest. Both WC-IC and WC-mid were measured to the nearest 0.1 cm at the end of a normal expiration. Before recording the measurement, the examiner ensured that the tape was snug and parallel to the floor but did not compress the skin.

Detailed medical history and complete neurological examinations were performed for all research participants by one of the authors (R.M.A., J.A., A.A., M.A.) following comprehensive training on the use of standard methods to document neuropathy symptoms and signs. Motor strength grading was performed using Medical Research Council criteria: 0 = no contraction, 1 = flicker or trace of contraction; 2 = active movement; with gravity eliminated; 3 = active movement against gravity; 4 = active movement against gravity and resistance; 5 = normal power [11]. A three-point score (2 = no abnormality; 1 = decrease; and 0 = absent) was used to assess muscle stretch reflexes. Decreased deep tendon reflexes are considered when the degree of contraction of a muscle after tapping its respective tendon (biceps, brachioradialis, triceps tendon insertions in the upper extremity or patellar and Achilles tendons in the lower extremity) is less than expected for a healthy person of similar age as judged by the examining physician. Absent reflexes are considered when no contraction of the muscle occurs after tapping the respective tendon and while applying reinforcement maneuvers such as the Jendrassik maneuver. Assessment of motor signs was performed on the right side. Three modalities of sensation (touch pressure, vibration, and pinprick) were tested at the distal toes on the right side and compared to the perception of each modality at the participant’s clavicle as a reference. If sensation was reduced or absent, testing was continued proximally in the extremity to determine the level of impaired sensation as follows: up to the ankle, mid-shin, knee, or above the knee. Joint position sense was tested at the distal phalanx of the right large toe; flexion or extension movements were performed passively by the examining physician while obscuring visual input by the participant. Joint position was graded as impaired if the participant responded with two or more errors in identifying the correct position of their joint. Heel and toe walking were assessed by asking participants to walk on their heels or toes, respectively, across a distance of 3 m on a horizontal floor and was considered impaired if the participant could not complete the task partially or completely. In cases with right hemiparesis or hemisensory impairment due to prior cerebrovascular stroke or hematoma, assessment for motor or sensory signs was performed on the left side.

### 2.2. Nerve Conduction Studies

NC studies were performed by one of the authors using standard methods (MI) [12]. Electromyography (Keypoint EMG system, Natus Industries, Middleton, WI, USA) with standard filter settings (2 Hz to 10 kHz), a surface stimulator, and round disk electrodes (10 mm diameter) was used to record nerve action potentials. All nerve conduction studies were performed on the right side with limb temperatures maintained above 31 degrees Celsius. Following supramaximal stimulation, fibular, tibial, median, and ulnar motor responses were recorded from the extensor digitorum brevis, abductor hallucis longus, abductor pollicis brevis, and abductor digiti minimi muscles, respectively. During the distal stimulation of motor nerves, the distance between the stimulator cathode and recording electrodes was maintained at 8 cm for the fibular and tibial motor nerve studies and 7 cm for the median and ulnar motor nerve studies. Ulnar and median antidromic sensory responses were recorded from digit 5 and digit 2, respectively, following a stimulus 14 cm proximal to the recording electrode. Sural sensory responses were recorded from the lateral foot following stimulation 14 cm proximal to the recording electrode. Studied nerve conduction attributes included the distal motor latency (DML) and sensory peak latency (SPL), compound muscle action potential (CMAP) and sensory nerve action potential (SNAP) amplitudes (both measured from baseline to negative peak), and motor nerve conduction velocity (MNCV) and sensory nerve conduction velocity (SNCV). The motor and sensory conduction velocities were calculated by dividing the measured distance by the onset latency. Normative reference values validated in the Saudi population using the criteria of the Normative Data Task Force of the American Association of Neuromuscular and Electrodiagnostic Medicine (AANEM) were used [13,14].

### 2.3. Laboratory Testing

Laboratory testing was performed for all research participants and included HbA1c, the lipid profile [high-density lipoprotein (HDL) (mmol/L), low-density lipoprotein (LDL) (mmol/L), triglycerides (TG) (mmol/L)], Cr (µmol/L), and the estimated glomerular filtration rate (eGFR) measured by the Chronic Kidney Disease Epidemiology Collaboration (CKD-EPI) formula [15]. Serum CysC (mg/L) levels were measured by particle-enhanced immunonephelometry using the BN ProSpec system (Siemens Healthineers, Forchheim, Germany). Urine albumin-to-creatinine ratio values were collected if tested. Nephropathy was defined as urine ACR > 30 mg/g. All laboratory values were obtained within 6 months of clinical evaluations and nerve conduction studies.

### 2.4. Definition of DM-DSPN and Severity Staging

We defined DM-DSPN clinically as the presence of symmetric large nerve fiber impairment (reduced or absent vibration sense, reduced or absent joint position sense, reduced or absent ankle stretch reflexes, weakness at the foot) in the lower extremity in a length-dependent gradient.

The staged severity of DM-DSPN was based on criteria described by Davies et al. [16] (Table 1).

In brief, the following NC attributes were assessed: fibular, ulnar, or tibial CMAPs; MNCVs, MNDLs, or sural SNAP; or sural SPLs. NC attributes with values occurring below the 3rd (CMAP or SNAP amplitudes, MNCVs) or above the 97th (DMLs or SPLs) percentile for normative values adjusted for age were considered abnormal.

The staged severity of DSPN was then determined based on (1) the presence or absence of NC criteria of DSPN as stated above, (2) the presence or absence of large-fiber neuropathy signs in the lower extremity (reduced or absent vibration, reduced or absent joint position, weakness at the foot, reduced or absent ankle stretch tendon reflex), and (3) the distribution pattern of neuropathy signs in the lower extremity. Each participant was assigned to one of the following severity stages: N0, N1, N2, or N3 (Table 1).

### 2.5. Definitions of Study Groups, Sample Size Estimation, and Recruitment

Diabetic persons with stage N2 or N3 are collectively designated as +DM/+DSPN. Diabetic participants with stage N0 are designated as diabetic persons without DSPN (+DM/−DSPN). Symptoms or signs of small-fiber neuropathy (i.e., sensation of pricking, stabbing, or burning pain or signs of impaired temperature or pain sensation or allodynia) were permitted to be present in both groups, +DM/+DSPN and +DM/−DSPN. As CysC is a marker of GFR and renal function, the hypothetical proportion of elevated CysC in either group was assumed based on the estimated prevalence of nephropathy in adult persons with type 2 diabetes in the Saudi population. The Saudi Diabetic Kidney Disease study [17] reported a 50% prevalence of nephropathy in 640 adults with type 2 diabetes according to eGFR values. Estimates of nephropathy in diabetic persons with or without large-fiber neuropathy defined by standard electrodiagnostic criteria are not available for our population, although studies in other populations show a significant correlation between the presence of diabetic DSPN and a lower eGFR, suggesting a lower prevalence of nephropathy in persons without DSPN [18,19]. Therefore, we assumed a hypothetical proportion of elevated CysC in +DM/−DSPN of 10% and a hypothetical proportion of elevated CysC in +DM/+DSPN of 50%. After setting a two-sided confidence level of 95% and statistical power of 80%, and incorporating the assumed hypothetical proportions, the sample size was calculated using an online calculator (OpenEpi, Version 3, open-source calculator). This indicated a minimum number of 21 participants in each group (+DM/−DSPN and +DM/+DSPN) to maintain a type 1 error below 5%.

### 2.6. Statistical Analysis

Normality of continuous variables was assessed using the Shapiro–Wilk test before analysis. For categorical variables, Fisher’s exact test was utilized to explore potential relationships, while the Mann–Whitney U test was applied to assess differences across groups in continuous variables. Logistic regression was employed to assess the covariates of DM-DSPN. Linear regression was used to examine variables associated with NC attributes and serum CysC levels.

Spearman rank correlation was used to analyze variables correlating with serum CysC levels. A receiver operating characteristic curve (ROC) analysis was employed to identify the optimal cut-off for serum CysC and other covariates that showed close associations with the presence of DM-DSPN. Sensitivity, specificity, and the area under the curve (AUC) were calculated to assess the diagnostic performance of these markers. Data analysis was conducted with RStudio (Version 4.2.2). *p* < 0.05 was considered statistically significant.

## 3. Results

### 3.1. Comparison of Baseline Characteristics and Laboratory Values in +DM/+DSPN and +DM/−DSPN

A total of 52 participants were included, 24 of whom met the criteria for +DM/+DSPN. The remaining 28 participants met the criteria for +DM/−DSPN. One participant from the +DM/−DSPN group was diagnosed with type 1 DM; all remaining participants were diagnosed with type 2 DM. From the +DM/+DSPN group, 16 participants (67%) met the criteria for severity stage N2 (abnormality of NCs and polyneuropathy signs in feet or legs) and eight participants (33%) met the criteria for severity stage N3 (abnormality of NCs and signs of thigh involvement). All participants in the +DM/−DSPN group met the criteria for stage N0 (no abnormality of NCs as assessed by composite NC score).

The median age of the +DM/+DSPN group was significantly higher (median = 63, IQR [56, 71]) than that of the +DM/-DSPN group (median = 58, IQR [52, 64]). +DM/+DSPN participants also exhibited significantly longer median diabetes duration (median = 20 years, IQR [12.3, 25]) compared to the +DM/−DSPN group (median = 15 years, IQR [6.5, 20]) (Table 2 and Table S1).

Physical examination revealed significant differences in various sensory and motor signs between groups. Pain sensation, vibration perception, and joint position were more frequently impaired at various distributions (up to ankle, mid-shin, at or above the knee) in +DM/+DSPN as compared to +DM/−DSPN (*p* < 0.05). Toe walking was more frequently impaired in the +DM/+DSPN group (*p* < 0.05) (Table 3). Two of the +DM/+DSPN group participants and four of the +DM/−DSPN participants had a prior stroke. In these participants, physical examination was performed on the left side to accurately assess motor strength and sensory findings if symptoms of stroke were on the right side (Supplementary Table S1). Laboratory testing did not show significant differences between the two groups, with the exception of higher HbA1c levels and lower platelet counts in the +DM/+DSPN group (Table S2).

### 3.2. Comparison of NC Attributes in +DM/+DSPN and +DM/−DSPN

All nerve conduction parameters were significantly different between groups, with the exception of median CMAP amplitudes and DMLs, median SPLs, and fibular DMLs. Overall, the +DM/+DSPN group exhibited reductions in SNAP or CMAP amplitudes, slowing MNCVs and SNCVs, and prolongation of DMLs and SPLs as compared to the +DM/−DSPN group (Supplementary Table S3).

### 3.3. Covariates Predicting DM-DSPN

Covariates thought to affect the risk of developing DSPN, such as age, gender, CysC levels, onset of diabetes, serum creatinine (Cr) levels, the serum glomerular filtration rate (GFR), hemoglobin A1c (HbA1c), and serum platelet levels, were subjected to univariate and multivariate analyses to assess potential associations with +DM/+DSPN. In the univariate regression, age (OR 1.07, 95% CI [1.01, 1.14]), onset of diabetes (OR: 1.08, 95% CI [1.01, 1.16]), serum CysC levels ≥ 0.88 mg/L (OR 13.5, 95% CI [3.08, 59.25]), and HbA1c ≥ 7.3 (OR: 4.37, 95% CI [1.35, 14.11]) were significantly associated with the presence of +DM/+DSPN. In the multivariate regression, only serum CysC ≥ 0.88 mg/L (OR: 12.88, 95% CI [1.58, 105.27]) and HbA1c ≥ 7.3 (OR: 16,33, 95% CI [2.28, 116.78]) remained statistically significant after controlling for Cr and GFR. Given the modest sample size and correlations among renal markers, we prioritized a parsimonious multivariable model; additional renal function-adjusted models were included for sensitivity analyses.

### 3.4. Receiver Operating Characteristic (ROC) and Area Under the Curve (AUC) Analysis for Covariates Associated with DSPN

**Figure 1:**
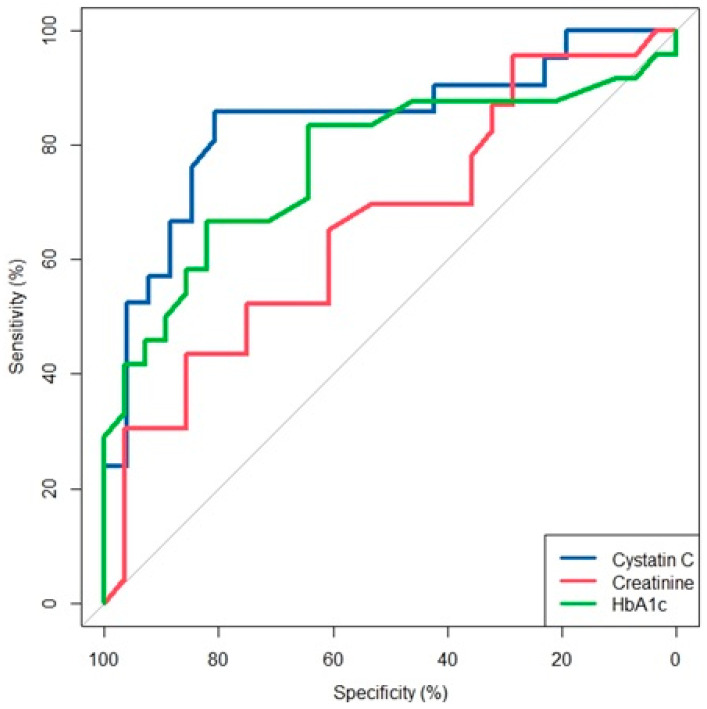
ROC/AUC plots of covariates associated with diabetic distal symmetric sensorimotor polyneuropathy.

The optimal cut-off point for CysC to show an association with the presence of +DM/+DSPN was 0.9 mg/L, exhibiting sensitivity of 86%, specificity of 81%, and an area under the curve (AUC) of 84%. For HbA1c, the optimal cut-off was 7.7, resulting in sensitivity of 67%, specificity of 82%, and an AUC of 77%. For serum Cr, the optimal cut-off point to show an association with DM-DSPN was 90 µmol/L, with sensitivity of 44%, specificity of 86%, and an AUC of 66% (Figure 1).

### 3.5. Associations of Serum CysC and NC Attributes

Linear regression was employed to identify factors associated with motor and sensory nerve conduction amplitudes. Covariates thought to affect various NC attributes were assessed and included age, gender, CysC levels, onset of diabetes, serum Cr levels, serum GFR levels, and HbA1c levels. In the multivariate regression, serum CysC levels were significantly associated with peroneal and ulnar motor amplitudes after controlling for GFR (Beta = −3.45, SE = 1.07, *p*-value = 0.003, Beta: −2.7, SE = 1.19, *p*-value = 0.03, respectively), but not sural or tibial motor amplitudes. Furthermore, serum CysC levels were significantly associated with peroneal and tibial MNCVs after controlling for GFR (Beta = −27.89, SE = 1.07, *p*-value = 0.009, Beta: −29.61, SE = 9.67, *p*-value = 0.0003, respectively), but not sural SNCVs or ulnar MNCVs. The optimal cut-off point for CysC in showing an association with ulnar CMAP amplitudes less than 4.8 mV was 1.7 mg/L, exhibiting sensitivity of 100%, specificity of 96%, and an AUC of 96%.

### 3.6. Covariates Associated with Cystatin C

CysC was significantly correlated with age (Rho = 0.60, *p*-value < 0.0001), serum Cr (Rho = 0.70, *p*-value < 0.0001), GFR (Rho = −0.74, *p*-value < 0.0001), and waist circumference (IC) (Rho = 0.38, *p*-value < 0.013). These variables retained significance in the univariate linear regression. However, in the multiple regression, only age, GFR, and Cr remained significant. To mitigate multicollinearity, we developed two separate multiple regression models, avoiding the simultaneous inclusion of GFR and Cr in the same model. No significant correlation was found between CysC and the onset of diabetes. No significant correlation was found between CysC and C-reactive protein (CRP) levels or the erythrocyte sedimentation rate (ESR).

## 4. Discussion

In this study, we show that serum CysC levels are independently associated with large-fiber DM-DSPN and specific NC attributes (peroneal and ulnar CMAP amplitudes, peroneal and tibial MNCVs) after controlling for GFR and HbA1c using validated and referenced diagnostic NC criteria for the diagnosis and staged severity of DM-DSPN [16]. Furthermore, our analysis reveals CysC to be a more sensitive covariate associated with the presence of DM-DSPN than HbA1c, with similar specificity (81% and 82%, respectively), irrespective of GFR or Cr levels.

The Neurodiabetes Consensus (Toronto) Group on diabetic peripheral neuropathies (2011) defines DM-DSPN as chronic, symmetric, length-dependent sensorimotor polyneuropathy developing from metabolic derangements and microvessel alterations related to chronic hyperglycemia and cardiovascular risk factors [20,21]. Nerve damage in DSPN affects the longest and most susceptible nerve fibers first and progresses distally to proximally, resulting in bilateral, symmetric, length-dependent sensory and motor deficits [21].

The duration of diabetes and HbA1c levels are major covariates of DM-DSPN [22]. Independently of glycemic status, symptomatic DM-DSPN is more common in persons with metabolic syndrome components, such as hypertriglyceridemia, hypertension, abdominal obesity, and low HDL levels [23]. The relationship between diabetic nephropathy and diabetic neuropathy is well established in medical literature. Low GFR is an independent risk factor for neuropathy, with studies showing an association between GFR and the presence of diabetic neuropathy independently of blood glucose, HDL, and patient demographics [17,24].

CysC is produced by all nucleated cells at a constant rate, freely filtered in the glomeruli, and almost entirely reabsorbed in the distal tubule [25]. CysC is considered an alternative or a more accurate estimator of GFR as compared to Cr [26], especially in patients with normal or mild-to-moderate renal dysfunction and is not affected by extra-renal factors such as age, sex, and muscle mass [27].

Xiong et al. [28] reported an association between serum CysC levels and various vascular outcomes (stroke, end-stage renal disease, myocardial infarction) or mortality (cardiovascular and all-cause). Moreover, CysC is a marker of inflammation and elevated systolic blood pressure [29]. The frequency of comorbid conditions such as hypertension, ischemic heart disease, and cerebrovascular stroke was similar in the +DM/+DSPN and +DM/−DSPN groups in our cohort. Serum levels of CRP and ESR, both considered markers of inflammation, were not different between groups in our study. Okura et al. found correlations between CysC and TNF-alpha or IL-6 but not with CRP. We did not test for TNF-alpha and IL-6 as these tests were not available in our laboratory at the time of participant enrollment [29].

**Figure 2:**
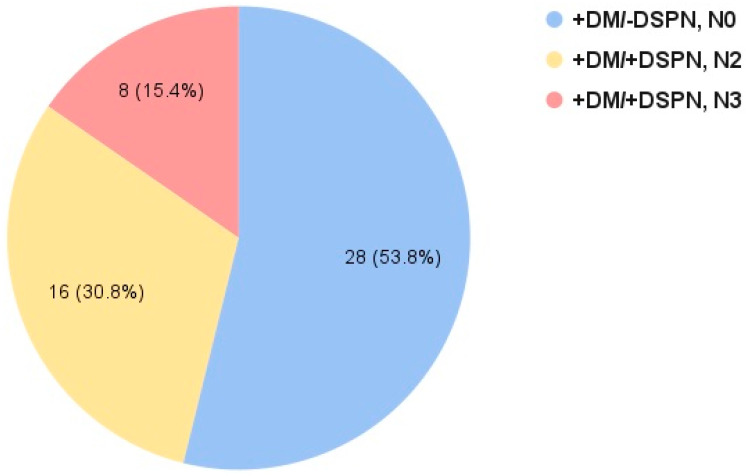
Staged severity of diabetic distal symmetric sensorimotor polyneuropathy in our cohort.

NC abnormalities precede clinical symptoms of DM-DSPN and are more sensitive for the detection of DSPN [20]. In their recent study, Davies et al. [16] validated criteria for the staged severity of DSPN using composite NC attributes (Table 1). The use of validated criteria for the definition and staged severity of DM-DSPN is crucial for the reproducibility and comparison of findings across clinical studies assessing the development and progression of DM-DSPN. Using the criteria validated by Davies et al. [16], the majority of the +DM/+DSPN group (67%) in our cohort was of severity stage N2 (abnormality of NCs and polyneuropathy signs in feet or legs), while the remaining 33% met the criteria for severity stage N3 (abnormality of NCs and signs of thigh involvement) (Figure 2).

In the study by Hu et al. [10], which included 937 diabetic persons, GFR levels in participants with only signs of DM-DSPN or with DM-DSPN confirmed by objective NC criteria were lower than those seen in diabetic persons without DSPN. Contrary to the findings of Hu et al., the Cr levels and GFR were similar in both groups in our cohort, while the CysC levels were significantly higher in the +DM/+DSPN group.

CysC is associated with cardiovascular disease independently of eGFR and was shown to predict stroke clinical outcomes at one year independently of renal function [30,31]. Moreover, a recent study showed that cystatin C (CysC) was independently associated with peripheral arterial disease in type 2 diabetes mellitus patients without overt nephropathy [32]. Further studies with larger numbers of persons with DM are needed to assess the variable effects on microvascular integrity in various end-organs (e.g., cardiac, retinal, peripheral nerve, renal) in relation to CysC levels.

Similarly to our findings, Hu et al. [10], found serum CysC levels to be significantly higher in persons with electrodiagnostic features of DM-DSPN as compared to persons with only signs of DM-DSPN and those without DM-DSPN. Additionally, in their cohort, CysC was associated with DM-DSPN after adjustment for age, diabetes duration, HbA1c, Cr, urinary albumin, and GFR. The cutoff point for serum CysC levels to have a close association with large-fiber DM-DSPN in our study (>0.9 mg/L) was slightly lower than that reported by Hu et al. [10], who reported a higher cut-off point for serum CysC in males as compared to females (1.25 mg/L vs. 1.05 mg/L, respectively) in showing an association with DM-DSPN.

We found significant associations between CysC and NC attributes in our cohort. CysC was associated with peroneal and tibial MNCVs and peroneal and ulnar CMAP amplitudes. Cystatin C levels above 1.7 had 100% sensitivity and 96% specificity in showing an association with ulnar CMAP amplitudes below 4.8 mV. To our knowledge, this is the first study to assess correlations between CysC and nerve conduction attributes in DSPN. NCS in our cohort were performed by a single experienced examiner applying population-specific normative reference values and validated NC criteria for defining DSPN. The findings of our study can guide future studies aimed at determining an exposure–response effect between serum CysC levels and varying severity stages of DSPN. Such findings could provide a basis for therapeutic trials targeting CysC to ameliorate the progression of DSPN.

Limitations of our study include the small sample size from a single tertiary hospital, which limits the statistical power and may increase the risk of overfitting in multivariable models. Accordingly, effect estimates, particularly those with wide confidence intervals, should be interpreted cautiously. The enrollment of diabetic persons in our study was not population-based, limiting the generalizability of the findings to community-based populations, patients with type 1 diabetes, and other ethnicities or healthcare settings.

## 5. Conclusions

The present study shows that, in persons with diabetes mellitus type 2, serum CysC levels >0.9 mg/L are associated with large-fiber DM-DSPN, with sensitivity and specificity of 86% and 81%, respectively, independently of renal function or glycemic control. CysC levels above 0.9 mg/L have higher sensitivity in showing an association with DM-DSPN compared to HbA1c (>7.7%), with similar specificity.

Serum CysC levels were also independently associated with peroneal and ulnar CMAP amplitudes, as well as peroneal and tibial MNCVs, after adjusting for GFR and HbA1c. Therefore, the serum CysC level may serve as a potential clinical biomarker of DM-DSPN, at least in type 2 diabetes. Our study is limited by the small sample size and retrospective nature; therefore, the cut-off points for CysC derived from the current analysis are exploratory in nature and not intended for clinical application. More rigorous longitudinal studies with larger numbers of participants, optimally inclusive of various stages of DM-DSPN severity, are required to establish the predictive validity of CysC in relation to DM-DSPN.

## Figures and Tables

**Table 1:** Staging severity of DSPN (Davies et al., 2023) [16].

Severity Stage	Description
N0	No abnormality of NCs ^§^ as assessed by composite NC score ^§^
N1	Abnormality of NC without neuropathy signs
N2	Abnormality of NCs and polyneuropathy signs in feet or legs
N3	Abnormality of NCs and signs of thigh involvement

NC = nerve conduction. ^§^ Abnormality of NCs defined as two or more NC attributes (fibular, ulnar or tibial CMAPs; MNCVs or MNDLs; or sural SNAP or SNDL) occurring below the 3rd (CMAP or SNAP amplitudes, MNCVs) or above the 97th (DMLs or SPLs) percentile for normative values adjusted for age in subjects of Saudi ethnicity.

**Table 2:** Demographic and clinical characteristics of patients with and without diabetic distal symmetric sensorimotor polyneuropathy (DM-DSPN).

Characteristic	Total N = 52	+DM/+DSPN N = 24	+DM/−DSPN N = 28	*p*-Value
Ethnicity (%)				
Saudi	50 (96)	23 (96)	27 (96)	1 ^ÿ^
Syrian	2 (4)	1 (4)	1 (4)	
Gender (%)				
Female	28 (54)	10 (42)	18 (64)	0.176 ^ÿ^
Male	24 (46)	14 (58)	10 (36)	
Age, median (IQR) years	60 (55, 66)	63 (56, 71)	58 (52, 64)	0.05 *^,†^
Duration of sensory symptoms in lower limbs, median (IQR) years	4.5 (2, 10)	6 (1.75, 10)	4 (2, 9.5)	0.65 ^†^
Pain, n (%)	37 (71.2)	16 (67)	21 (75)	0.723 ^ÿ^
Prickling, n (%)	21 (40)	12 (50)	9 (32)	0.305 ^ÿ^
Paresthesia, n (%)	24 (46)	13 (54)	11 (39)	0.427 ^ÿ^
Numbness, n (%)	33 (64)	16 (67)	17 (61)	0.876 ^ÿ^
Weakness in lower extremities, n (%)	10 (19)	6 (25)	4 (14)	0.532 ^ÿ^
Dysphagia, n (%)	4 (8)	3 (13)	1 (4)	0.495 ^ÿ^
Orthostatic lightheadedness, n (%)	21 (40)	11 (46)	10 (36)	0.647 ^ÿ^
Heat intolerance, n (%)	9 (17)	4 (17)	5 (18)	1 ^ÿ^
Male impotence, n (%)	4 (8)	4 (17)	0	0.084 ^ÿ^
Sphincter dysfunction, n (%)	19 (37)	13 (54)	6 (21)	0.031 *^,ÿ^
Early satiety, n (%)	10 (19)	7 (29)	3 (11)	0.183 ^ÿ^
Onset of diabetes, median (IQR) years	15 (9, 20.5)	19.5 (12.3, 25)	15 (6.5, 20)	0.044 *^,†^
Diabetes type, n (%)				
Type 1	1 (2)	0	1 (4)	1 ^ÿ^
Type 2	50 (98)	23 (100)	27 (96)	

DM = diabetes mellitus; +DM/+DSPN = diabetic patients with stage N2 or N3 DSPN; +DM/−DSPN = diabetic persons without DSPN; DSPN = distal symmetric sensorimotor polyneuropathy; ^ÿ^ Fisher’s exact test; * Statistically significant (≤0.05); ^†^ Mann-Whitney U test.

**Table 3:** Anthropometric and lower-extremity neurological examination findings in diabetic distal symmetric sensorimotor polyneuropathy.

	Total N = 52	+DM/+DSPN N = 24	+DM/−DSPN N = 28	*p*-Value
Anthropometric values, median (IQR)				
Height, cm	160 (153, 169)	161 (152, 169)	160 (154, 169)	0.993 ^†^
Weight, kg	77 (65, 86)	71 (64, 86)	79 (67, 86)	0.707 ^†^
BMI kg/m^2^	29 (26, 33)	29 (26, 31)	28 (26, 3)	0.993 ^†^
Waist circumference (WC-IC), cm	105 (93, 111)	110 (93, 115)	97 (94, 109)	0.166 ^†^
Waist circumference (WC-mid), cm	98 (89, 109)	101 (91, 115)	94 (87, 103)	0.136 ^†^
Sensory examination				
Pinprick, n (%)				
Normal	12 (23)	2 (8)	10 (36)	0.002 *^,ÿ^
Hyperalgesia	1 (2)	1 (4)	0	
Reduced up to ankle	21 (40)	6 (25)	15 (54)	
Reduced up to mid-shin	12 (23)	9 (38)	3 (11)	
Reduced up to knee	4 (8)	4 (17)	0	
Reduced to above knee	2 (4)	2 (8)	0	
Vibration, n (%)				
Normal	12 (23)	3 (13)	9 (32)	0.014 *^,ÿ^
Reduced up to ankle	18 (35)	5 (21)	13 (46)	
Reduced up to mid-shin	1 (2)	1 (4)	0	
Reduced up to knee	8 (15)	7 (29)	1 (4)	
Reduced to above knee	13 (25)	8 (33)	5 (18)	
Impaired joint position at large toe, n (%)	11 (21)	10 (42)	1 (4)	0.003 *^,ÿ^
Impaired heel walking, n (%)	5 (10)	4 (17)	1 (4)	0.261 ^ÿ^
Impaired toe walking, n (%)	7 (14)	7 (30)	0	0.008 *^,ÿ^
Ankle stretch reflex, n (%)				
Absent	21(40)	14 (58)	7 (25)	0.035 ^ÿ^
Reduced	6 (12)	1(4)	5 (18)	
Normal	25 (48)	9 (38)	16 (57)	

cm = centimeters; +DM/+DSPN = diabetic patients with stage N2 or N3 DSPN; +DM/−DSPN = diabetic persons without DSPN; IQR = interquartile range; WC-IC = waist circumference—iliac crest; WC-mid = waist circumference—midway. * Statistically significant (≤0.05); ^†^ Mann–Whitney U test; ^ÿ^ Fisher’s exact test.

## Data Availability

The data that support the findings in our research are available from the corresponding author upon reasonable request.

## Supplementary Materials

The following supporting information can be downloaded at https://www.mdpi.com/article/10.3390/biomedicines14030544/s1. Table S1. Other demographic and clinical characteristics of patients with and without diabetic distal symmetric sensorimotor polyneuropathy (DM-DSPN); Table S2. Laboratory values in diabetic distal symmetric sensorimotor polyneuropathy; Table S3. Nerve conduction values in diabetic distal symmetric sensorimotor polyneuropathy.

## Abbreviations

BMI = body mass index; CMAP = compound muscle action potential; CKD-EPI = Chronic Kidney Disease Epidemiology Collaboration; Cr = creatinine; CRP = C-reactive protein; CysC = cystatin C; DBP = diastolic blood pressure; DML; distal motor latency; DM = diabetes mellitus; DM-DSPN = diabetic distal symmetric sensorimotor polyneuropathy; DSPN = distal symmetric sensorimotor polyneuropathy; EMG = electromyography; ESR = erythrocyte sedimentation rate; GFR = glomerular filtration rate; HbA1c = hemoglobin A1c; HDL = high-density lipoprotein; IHD = ischemic heart disease; LDL = low-density lipoprotein; MI = myocardial infarction; MNCV = motor nerve conduction velocity; mV = millivolt; NC = nerve conduction; NCS = nerve conduction study; SBP = systolic blood pressure; SNCV = sensory nerve conduction velocity; SPL = sensory peak latency; TG = triglyceride; WC-IC = waist circumference—iliac crest; WC-mid = waist circumference—mid-abdomen; WBC = white blood cells.
