# Supplementary material for: Clinical Utility of Serum Cystatin C in Predicting Diabetic Distal Sensorimotor Polyneuropathy
Source: Biomedicines. 2026 Feb 27;14(3):544. doi: 10.3390/biomedicines14030544 (PMC13023794; doi:10.3390/biomedicines14030544)
Supplement: Supplementary file 1 [file biomedicines-14-00544-s001.zip › biomedicines-4118179-supplementary.pdf]

Table S1**.** Other Demographic and clinical characteristics of patients with and without diabetic distal symmetric sensorimotor polyneuropathy (DM-DSPN).

Characteristics	Total<br>N = 52	+DM/+DSPN<br>N = 24	+DM/-DSPN<br>N = 28	P-Value
Medications, n (%)				
Metformin	42 (81)	18 (75)	24 (86)	0.532[ÿ]
Sulfonylureas	17 (33)	6 (25)	11 (39)	0.425[ÿ]
GLP 1 agonists	4 (8)	3 (13)	1 (4)	0.495[ÿ]
Pioglitazone	2 (4)	1 (4)	1 (4)	1[ÿ]
DPP4 inhibitors	19 (37)	11 (46)	8 (29)	0.317ÿ
Insulin	20 (39)	15 (63)	5 (18)	0.003[*ÿ]
Thiazolidinediones	2 (4)	2 (8)	0	0.404[ÿ]
Atorvastatin	14 (27)	4 (17)	10 (36)	0.171[ÿ]
Simvastatin	1 (2)	0	1 (4)	
Diabetic nephropathy, n /total tested[§]	2/50	1/22	1/28	1[ÿ]
Diabetic retinopathy, n (%)	11 (21)	7 (29)	4 (14)	0.332[ÿ]
Hypertension, n (%)	28 (55)	14 (61)	14 (50)	0.622[ÿ]
Comorbid conditions, n (%)				
IHD	3 (6)	3 (13)	0	0.203[ÿ]
Stroke	5 (10)	2 (9)	3 (11)	
Stroke and IHD	1 (2)	0	1 (4)	
Smoking, n (%)				
Second hand	3 (6)	1 (4)	2 (7)	0.858[ÿ]
Ex- smoker	3 (6)	2 (8.3)	1 (4)	
Current	6 (12)	3 (13)	3 (11)	
Alcohol consumption, n (%)	1 (2)	0	1 (4)	1[ÿ]

DM=Diabetes mellitus; +DM/+DSPN= Diabetic patients with stage N2 or N3 DSPN; +DM/-DSPN= diabetic persons without DSPN; DSPN=distal symmetric sensorimotor polyneuropathy; DPP4=dipeptidyl peptidase 4; GLP-1=glucagon-like peptide-1; IHD=ischemic heart disease. [§]Diabetic nephropathy defined as albumin-to-creatinine ratio (ACR)>30mg/g [*] Statistically significant (<= 0.05); [ÿ] Fisher-exact test.

Table S2. Laboratory values in diabetic distal symmetric sensorimotor polyneuropathy

Laboratory value,<br>median (IQR)	Overall<br>N = 52	+DM/+DSPN<br>N = 24	+DM/-DSPN<br>N = 28	P-value

WBC, microliter	6.8 (5.1, 8.2)	7.5 (5,8)	6.1 (4.8, 7.5)	0.255†
Hb, g/dl	12.9 (12.2, 14.1)	12.8 (11.8, 13.3)	13.3 (12.4, 15.2)	0.093†
PLT, × 10^9/L	255 (212, 305)	238 (207, 273)	272 (215, 333)	0.041*†
Creatinine, µmol/L	72(57, 91)	82 (60, 100)	66 (52, 83)	0.048*†
GFR, ml/min/1.73 m^2‡	89 (76, 102)	81 (65, 99)	92 (81, 103)	0.066†
AST, U/L	17 (13, 22)	16 (13, 23)	17 (13, 21)	0.898†
ALT, UL	23 (17, 34)	25 (17, 31)	22 (18, 35)	0.862†
GGT, U/L	26 (18, 38)	28 (22, 38)	21 (16, 36)	0.225†
TSH, mIU/L	1.5 (1.2, 3.1)	1.5 (1.1, 3.3)	1.5 (1.3, 2.6)	0.888†
FT4, pmol/L	15.3 (14, 16.5)	15.1 (14.1, 16)	15.6 (14.1, 17)	0.39†
HbA1C (%)	7.3 (6.5, 9)	8.7 (7.1, 9.7)	6.7 (6.3, 7.5)	0.001*†
Cholesterol, mmol/L	4.3 (3.5, 5.1)	3.9 (3.4, 5.1)	4.5 (4, 5.1)	0.201†
Triglycerides, mmol/L	1.2 (1, 1.4)	1.1 (1, 1.4)	1.2 (1, 1.4)	0.857†
LDL, mmol/L	2.5 (1.7, 3.1)	1.9 (1.5, 2.9)	2.6 (2.2, 3.1)	0.061†
HDL, mmol/L	1.2 (1, 1.5)	1.1 (1, 1.4)	1.3 (1.1, 1.6)	0.112†
Vitamin B12, pmol/L	299 (218, 459)	270 (205, 355)	369 (240, 519)	0.067†
Cystatin C, mg/L	0.9 (0.7, 1.1)	1.1 (1, 1.3)	0.7 (0.6, 0.9)	<0.001*†
Pre-Beta lipoprotein, mg/dL	18 (13, 23)	18 (15, 23)	18 (13, 24)	0.951†
Beta-lipoprotein, mg/dL	47 (42, 53)	45 (42, 54)	48 (42, 53)	0.951†
Alpha-lipoprotein, mg/dL	34 (28, 40)	33 (26, 41)	35 (32, 39)	0.355†
CRP, mg/dL	1.9 (1.1, 4)	1.7 (1.2, 3)	2.2 (0.6, 5)	0.861†
ESR, mm/hour	27 (14, 39)	28 (16, 38)	25 (14, 40)	0.733†

+DM/+DSPN= Diabetic patients with stage N2 or N3 DSPN; +DM/-DSPN= diabetic persons without DSPN; IQR=interquartile range; WBC=White blood cells; Hb=Hemoglobin, PLT=platelets; GFR=glomerular filtration rate; AST=Aspartate aminotransferase; ALT=alanine aminotransferase; GGT=Gamma-glutamyl transpeptidase; TSH; thyroid-stimulating hormone; FT4=free thyroxine; LDL=low-density lipoprotein; HDL=high-density lipoprotein; CRP=C-reactive protein; ESR=erythrocyte sedimentation rate.

* Statistically significant (<= 0.05); † Mann-Whitney U test

‡GFR was calculated using CKD-EPI (Chronic Kidney Disease Epidemiology Collaboration) equation(18)

Table S3. Nerve conduction values in diabetic distal symmetric sensorimotor polyneuropathy.

	**Overall N = 52**	**+DM/+DSPN N = 24**	**+DM/-DSPN N = 28**	**P-Value**
**Motor CMAP amplitudes, median (IQR) millivolts**				
**Fibular**	3.2 (0.9, 4)	0.8 (0.2, 3)	4.3 (3, 5)	<0.001*†
**Tibial**	6.5 (3, 9)	2.6 (0.9, 5)	9.1 (8, 12)	<0.001*†
**Median,**	8.4(7, 9)	7.5 (6, 9)	8.8 (7, 10)	0.068†
**Ulnar**	8.9 (7, 10)	7.4 (7, 8)	9.5 (9, 10)	<0.001*†
**Sensory SNAP amplitudes, median (IQR) microvolts**				
**Ulnar**	20.9 (14, 32)	14.7 (7, 20)	27 (21, 41)	<0.001*†
**Median**	14.6 (6, 26)	6.7 (5, 15)	21 (14, 42)	0.001*†
**Sural**	6.4 (0.4, 12)	0 (0, 5)	11 (8, 14)	<0.001*†
**Motor nerve conduction velocities, median (IQR) meters/second**				
**Fibular**	44.5 (38, 49)	36.8 (11, 42)	48.1 (46, 50)	<0.001*†
**Tibial**	45.2 (41, 50)	40.2 (32, 45)	49.2 (45, 51.6)	<0.001*†
**Median**	22 (12, 30)	15 (10, 22)	28.5 (17, 37)	<0.001*†
**Ulnar**	54.5 (51, 57)	51.9 (48, 54)	55.3 (53, 61)	0.001*†
**Sensory nerve conduction velocities, median (IQR) meters/second**				
**Ulnar**	57.3 (51, 62)	51.2 (46, 58)	60.4 (57, 63)	0.002*†
**Median**	45.9 (34, 53)	39.8 (31, 50)	48.7 (41, 60)	0.025*†
**Sural**	48.5 (9, 54)	0 (0, 43)	53.1 (49, 59)	<0.001*†
**Distal motor latencies, median (IQR) milliseconds**				
**Fibular**	4.2 (3.6, 4.6)	4.5 (1.42, 5.13)	4.1 (3.7, 4.4)	0.691†
**Tibial**	3.9 (3.3, 4.4)	4.5 (4, 5)	3.67 (3.2, 3.9)	0.004*†
**Median**	3.9 (3.5, 5.1)	4.3 (3.6, 5.1)	3.8 (3.1, 4.7)	0.15†
**Ulnar**	2.9 (2.6, 3.1)	3.1 (2.8, 3.3)	2.7 (2.5, 2.9)	<0.001*†
**Sensory peak latencies, median (IQR) milliseconds**				
**Ulnar**	3 (2.7, 3.3)	3.2 (3, 3.5)	2.7 (2.6, 3)	<0.001*†
**Median**	3.6 (2.9, 4.5)	3.8 (3.3, 4.9)	3.5 (2.8, 4)	0.142†
Sural	3.3 (0.7, 3.5)	0 (0, 3.5)	3.4 (3.1, 3.6)	0.012*†

+DM/+DSPN= Diabetic patients with stage N2 or N3 DSPN; +DM/-DSPN= diabetic persons without DSPN; IQR=interquartile range. * Statistically significant (<= 0.05); † Mann-Whitney U test.
